# Redox Regulation of NOX Isoforms on FAK^(Y397)^/SRC^(Y416)^ Phosphorylation Driven Epithelial-to-Mesenchymal Transition in Malignant Cervical Epithelial Cells

**DOI:** 10.3390/cells9061555

**Published:** 2020-06-26

**Authors:** Young Mee Kim, Karthika Muthuramalingam, Moonjae Cho

**Affiliations:** 1 Department of Biochemistry, School of Medicine, Jeju National University, Jeju 690-756, Korea; karthidol@gmail.com; 2 Institute of Medical Science, Jeju National University, Jeju 690-756, Korea; 3 Interdisciplinary Graduate Program in Advanced Convergence Technology & Science, Jeju National University, Jeju 690-756, Korea

**Keywords:** NAPDH oxidase, TGF-β1, pSRC (Y416), Sirt1, integrins

## Abstract

Epithelial-to-mesenchymal transition (EMT) promulgates epithelial cell associated disease-defining characteristics in tumorigenesis and organ fibrosis. Growth factors such as epidermal growth factor and fibroblast growth factor in addition to cytokines such as transforming growth factor-β1 (TGF-β1) is said to play a prominent role in remodeling related pathological events of cancer progression such as invasion, metastasis, apoptosis, EMT, etc. through redox related cellular secondary messengers, in particular the reactive oxygen species (ROS). However, the signaling cascade underlying the redox mechanism and thereby the progression of EMT remains largely unknown. In this study, upon TGF-β1 treatment, we observed an induction in NOX isoforms—NOX2 and NOX4—that have time (early and late) and cellular localization (nucleus and autophagosome co-localized) dependent effects in mediating EMT associated cell proliferation and migration through activation of the focal adhesion kinase (FAK)/SRC pathway in HeLa, human cervical cancer cells. Upon silencing NOX2/4 gene expression and using the SRC inhibitor (AZD0530), progression of TGF-β1 induced EMT related cellular remodeling, extra cellular matrix (ECM) production, cell migration and invasion, got significantly reverted. Together, these results indicate that NOX2 and NOX4 play important, albeit distinct, roles in the activation of cytokine mediated EMT and its associated processes via tyrosine phosphorylation of the FAK/SRC pathway.

## 1. Introduction

Cervical cancer, the third most common tumor among the female population, is only second to breast cancer in terms of incidence [1]. Poor prognosis and therapeutic outcome associated with early metastasis of primary tumors relating to the cervical cancer progression is of great concern. Beyond the realm of developmental biology, epithelial-to-mesenchymal transition (EMT) is recently receiving much importance in two more broad spectra of biology: cancer metastasis and organ fibrosis. By making the epithelial cells lose their polarity by modulating the deposition of extra cellular matrix (ECM) components, cell–cell junction proteins, cellular migration and invasion, apoptosis resistance, etc., this EMT process is controlled by numerous transcription factors and messenger molecules [2,3]. Despite extensive analysis of transcription factors and EMT modulators, the precise mechanisms underlying EMT progression remains largely unclear and is of much intrigue.

Notably, many key EMT regulators were recently found to be redox-sensitive, enabling the elucidation of the molecular basis of EMT from a redox perspective. Through these redox modifications, ROS, an important cellular secondary messenger containing free radical species such as superoxide anion (O_2_^−^) and hydrogen peroxide (H_2_O_2_), can alter the biological functions of redox-sensitive proteins involved in extracellular matrix (ECM) remodeling and cell mobility, thereby regulating EMT [4,5]. One of the primary sources of ROS production is via NADPH oxidase (NOX) enzyme, a family of heme-containing proteins with the primary function of transporting electrons from NADPH to oxygen, forming ROS [6]. Previous studies have indicated that NOX-dependent ROS production can alter cell motility or potentiate metastatic progression [7].

Studies have found that growth factors including transforming growth factor-β1 (TGF-β1), epidermal growth factor, platelet-derived growth factor, and hepatocyte growth factor induce cellular morphological changes characterized by the loss/gain of epithelial/mesenchymal markers [8,9,10]. In particular, such factors control the expression of Twist1, ZEB2 (SIP1), snail, slug, vimentin, and YB-1 during the induction of EMT by activating cell signaling pathways such as TGF-β1/Smad, PI3K/AKT, and MEK/ERK [11]. As a multifunctional cytokine, TGF-β1 can promote diverse cellular events including cell cycle arrest, survival, proliferation, and differentiation by upregulating targets such as the CDK inhibitors p21 or p27 [12]. This dimeric cytokine is aberrantly secreted in tumor stroma, consisting of large array of tumor-infiltrating cells such as cancer-associated fibroblasts, inflammatory cells, etc., which collectively co-ordinate in the regulation and progression of tumors [13,14,15]. It is well known that ECM remodeling and cell–cell junctions are altered in accordance with canonical and non-canonical TGF-β pathways, both of which are affected by cellular redox status [16]. In particular, TGF-β1-induced NOX4 has been implicated in osteoblast differentiation, fibroblast proliferation, endothelial cell cytoskeletal rearrangement, cell motility, EMT, and pulmonary fibrosis [17,18,19,20]. Although NOX2, also termed gp91phox, is mainly expressed in phagocytes, the upregulation of this NOX isoform and EMT were inhibited in TGF-β1-knockout cells, suggesting that TGF-β1 is required for NOX2 activation during renal fibrosis and in vascular endothelial cells [21,22].

Tyrosine kinases such as Src and focal adhesion kinase (FAK) were implicated widely in a range of processes such as cell cycle, cellular migration, proliferation, cell differentiation, cytoskeletal organization, etc. [23]. It was previously reported that Integrin and Src cooperate with TGF-β1 to induce mammary epithelial cell EMT. Additionally, TGF-β stimulation induces phosphorylation of the cytoplasmic domain of integrin, resulting in its activation and tumor cell invasion, survival, proliferation, and migration. Integrins have also been shown to control NADPH oxidase, as well as mitochondrial ROS production, via interactions with small G proteins [24,25,26,27]. Amidst several studies, yet the mechanisms through which redox modifications affect Src, FAK, or integrins which are involved in EMT and its associated cellular modulations are less well understood.

Previously, we investigated the involvement and role of NOX in ROS-mediated EMT in human HeLa cervical cancer cells [28,29]. In this present study, we intended to further expand our study by analyzing the spatial-temporal distribution of NOX isoforms and its impact on redox-dependent mechanisms involving Src, FAK, integrins, MAPK enzymes, etc., in the TGF-β1-induced EMT process in HeLa cervical cancer cells.

## 2. Materials and Methods

### 2.1. Cell Culture and Treatment

HeLa cells (American Type Culture Collection CCL-2, Manassas, VA, USA) were cultured in Dulbecco’s modified Eagle medium (DMEM, Corning, Armonk, NY, USA) supplemented with 10% fetal bovine serum (FBS; Gibco Inc., Grand Island, NY, USA). For each experiment, cells were untreated or treated with TGF-β1 (5 ng/mL, Invitrogen, Carlsbad, CA, USA), DPI (5 µM, Sigma-Aldrich, St. Louis, MO, USA), NAC (10 mM, Sigma-Aldrich, St. Louis, MO, USA), and apocynin (10 µM, Sigma-Aldrich). The treatment times were either 24 or 48 h depending on the particular assay. Each experiment was conducted at least three times.

### 2.2. Determination of ROS Generation

Intracellular ROS levels were determined using 2′,7′–dichlorofluorescein diacetate (DCFDA) assay. After respective treatment for the indicated period, the cells were incubated with DCFDA (10 μΜ) in complete medium for 30 min at 37 °C. As a positive control, 1 mM H_2_O_2_ diluted in PBS was added. The emitted fluorescence was read in a microplate spectrophotometer plate reader (GENios, TECAN Group, Maennedorf, Switzerland) at Ex/Em 502/535 nm.

### 2.3. MTT Assay

Cell viability/cytotoxicity was determined using a 3-(4, 5-dimethyl-2-thiazolyl)-2, 5-diphenyl-2H-tetrazolium bromide (MTT) assay). Cells were seeded into 96-well culture plates at a density of 3000 cells per well and then treated with TGF-β1. After different times of incubation, the assay was performed by adding 20 µL of MTT solution (5 mg/mL in PBS; Sigma-Aldrich) to each well, followed by incubation for 4 h. The crystals were dissolved with 200 µL of dimethyl sulfoxide and the formazan crystal production was quantified using a spectrophotometer (562 nm).

### 2.4. Cell Proliferation Based on 5-Bromodeoxyuridine (BrdU) Incorporation

To analyze cell proliferation, 5-bromo-2′-deoxyuridine (BrdU), a chemical analog of thymidine, was used. Briefly, cells seeded in black 96-well plates were incubated overnight followed by replacement of the medium with or without TGF-β1. At a final concentration of 10 μM, BrdU labeling solution was added and the cells were incubated for different times. At the end of indicated period, cells were fixed, which was followed by anti-BrdU antibody incubation for 1 h. After washing with 1× PBS solution, chemiluminescence was read at 450 nm using a Luminance reader (GENios, TECAN Group).

### 2.5. Western Blotting and Antibodies

Cells were washed twice with PBS and harvested in RIPA buffer (with 1 mM phenylmethylsulfonyl fluoride, Biosesang, Seongnam, Gyeonggi, Korea). Total protein amounts were measured using the bicinchoninic acid assay (Pierce, Rockford, IL, USA). Appropriate amounts of protein were subjected to sodium dodecyl sulfate-polyacrylamide gel electrophoresis (10–12% gels) and transferred to polyvinylidene fluoried membranes. Membranes were blocked with 10% nonfat dry milk in TBST (50 mM Tris/HCl and 150 mM NaCl)/0.1%Tween 20 and incubated with primary antibodies overnight. The primary antibodies used included those against Smad2/3, Zo-1, ZEB1, Cyclin E, Cyclin D, Keratin 14, EEA1, p67phox, p47phox (Santa Cruz Biotechnology, Dallas, TX. USA), slug, snail, vimentin, AKT, pAKT, p38, pp38, ERK, pERK, pSmad2, pSmad3, p21, p27, Src, LC3, pSrc (Y416), p40phox, pp40phox, GAPDH (Cell Signaling Technology, Beverly, MA, USA), ZEB2, YB-1, FAK, pFAK(Y397) (Abcam, Cambridge, UK), NOX4 (Novus Biologicals, Littleton, CO, USA), and NOX2 (BD Biosciences, San Diego, CA, USA).

### 2.6. Reverse Transcription Polymerase Chain Reaction (RT-PCR)

Total RNA was prepared from cells using TRI reagent (MRC, Cincinnati, OH, USA) according to the manufacturer’s instructions. Reverse transcription was performed using the Reverse Transcription System (Promega, Madison, WI, USA). PCR analyses were carried out with specific primer sets indicated in Table 1, which was followed by analyzing the samples by electrophoresis with 1% agarose gels containing 0.002% nucleic acid staining solution (TopRed, Biopure, United Kingdom).

### 2.7. In Vitro Wound Healing Assay

Migration was assessed using a wound healing assay. Cells were seeded in six-well plates and allowed to grow for three days to confluence. At time 0, media were removed, and cells were wounded using a 200 μL pipette tip. Cell debris was washed off twice with PBS, fresh media was added, and plates were returned to be incubated at 37 °C for 48 h. Phase contrast light microscope images were taken under a phase-contrast inverted microscope immediately after scratch wounding (0 h) and at 24 and 48 h. All experiments were repeated three times. The results were quantitated from the photographs using Image J software (National Institutes of Health, Bethesda, MD, USA).

### 2.8. Invasion Assay

HeLa cells were serum-starved with DMEM containing 1% FBS overnight. Cells (6 × 10^4^/well) were seeded on Matrigel-coated and uncoated Transwell inserts with a 8-μm pore size and then transferred to the lower chamber in a 24-well plate containing medium supplemented with 2% FBS (negative control) with/without 5 ng/mL TGF-β1 (stimulant, positive control). The wells were incubated for 24 h in a 37 °C CO_2_ incubator and the Transwell inserts were then removed from the 24-well plate. Remaining cells that had not migrated from the top of the insert were removed with wet cotton swabs and samples were fixed with 4% paraformaldehyde for 10 min and stained with crystal violet.

### 2.9. siRNA Transfection

Chemically synthesized RNA, purchased from Bioneer (Daejeon, Korea), was used, and annealed according to the manufacturer’s protocol. HeLa cells were grown to 50% confluence in 100-mm dishes and transfected with 20 nM control or validated NOX2 siRNA or NOX4 siRNA using OmicsFect (Omicsbio, Taipei City, Taiwan). Cells were used for experiments 48 h after transfection.

### 2.10. Electric Cell Substrate Impedance Sensing Assay

For impedance measurements, an ECIS instrument model Z (Applied Biophysics, Troy, NY, USA) was used. To start the trials, electrodes (pre-treated with aqueous 10 mM cysteine solution as recommended by the manufacturer) were filled with 400 µL of DMEM. HeLa cells were seeded at 1 × 10^4^ cells/well on electrode plates, and after a 24 h incubation, cells were allowed to grow in 2% FBS-containing medium containing 5 ng/mL TGF-β1. The entire electrode array was placed in a plate holder connected to the ECIS system and cell proliferation analysis was performed using the ECIS system software (Applied Biophysics). A growth curve was plotted based on cell proliferation percentage and time.

### 2.11. Immunofluorescence

Cells grown on Lab Tek eight-well chamber slides at an initial cell seeding density of 2 × 10^5^ cells/well were fixed with 4% buffered paraformaldehyde in PBS and washed three times with PBS. Fixed cells were then permeabilized with 0.3% triton X-100 and incubated in 5% bovine serum albumin for 1 h. Samples were incubated with the primary antibody overnight at 4 °C and subsequently incubated with a secondary antibody conjugated with Alexa Fluor 595, Alexa Fluor 488, or Texas red for 1 h at room temperature. After several washes with PBS, the slides were incubated with DAPI for 5 min and then mounted in glycerol. Images were photographed and analyzed using an EVOS system (Advanced Microscopy Group, Bothell, WA, USA).

### 2.12. Statistical Analysis

All experiments were performed in triplicate and repeated three times. Statistical analyses were performed using Excel (Microsoft, Redmond, WA, USA) and GraphPad Prism version 6.0 (GraphPad Software, La Jolla, CA, USA). Data were expressed as the means ± standard deviations (SDs). Values of *p* < 0.05 were considered to indicate statistical significance.

## 3. Results

### 3.1. NAPDH Oxidase Isoforms—NOX2 and NOX4—Regulates EMT and Cell Migration in TGF-β1-Treated HeLa Cells

**Figure 1:**
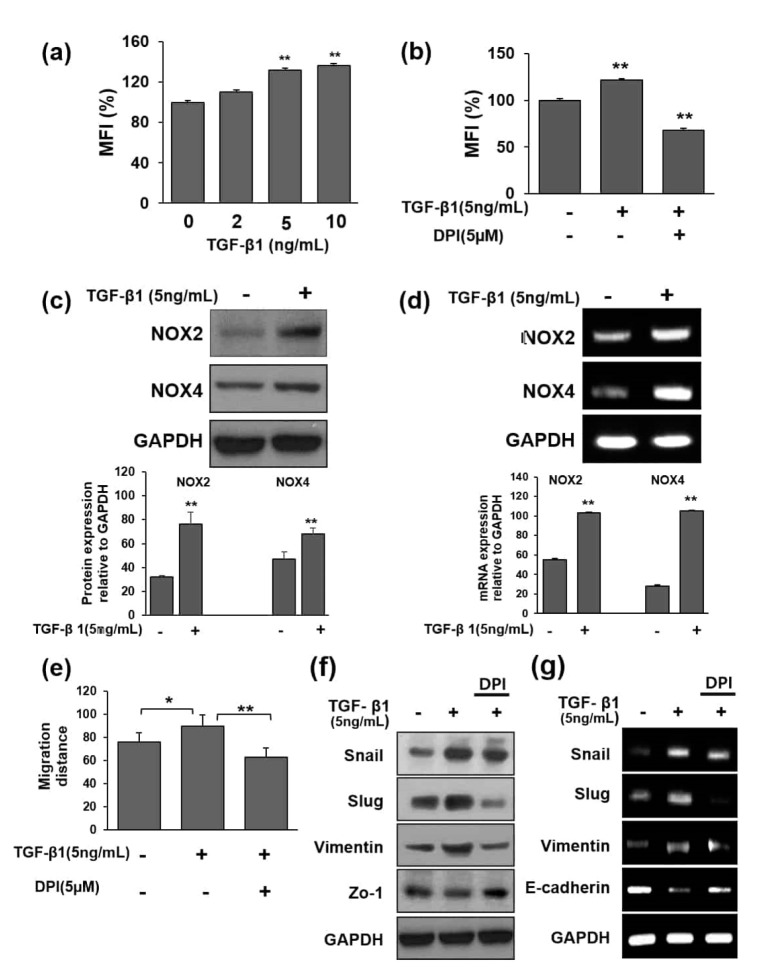
Transforming growth factor-β1 (TGF-β1) induces NOX2 and NOX4-dependent reactive oxygen species (ROS) generation in HeLa cells. (**a**) ROS levels in treated HeLa cells were measured by performing DCFDA assay. Cells were treated with varying concentrations of TGF-β1 for 24 h and then stained with DCFDA to detect ROS generation (MFI: median fluorescent intensity of DCFDA fluorescence). (**b**) Effect of TGF-β1-induced ROS generation. Cells were pretreated with 5 μM DPI 1 h before TGF-β1 stimulation for 24 h. Fluorescence was quantified using TECAN GENIous. (**c**,**d**) HeLa cells were treated with TGF-β1 for 24 h. The expression of NOX2 and NOX4 was examined by Western blotting and RT-PCR. GAPDH was used as a loading control. (**e**) Scratch wound healing assay of HeLa cells treated with TGF-β1 for 24 h, with results presented relative to those of control cells. Cells were seeded at a density of 3 × 10^4^ cells/mL 24 h prior to scratching and treatment. The areas of scratches were measured after treatment with TGF-β1 for 24 h. DPI (5 μM) was administered 1 h before the addition of 5 ng/mL TGF-β1. (**f**) Epithelial-to-mesenchymal transition (EMT)-related proteins in HeLa cells treated with TGF-β1. Cells were treated with TGF-β1 for 24 h. Protein lysates were then obtained from TGF-β1-treated cells using RIPA buffer and analyzed by Western blotting for snail, slug, vimentin, and ZO-1 expression. GAPDH was used as a loading control. (**g**) Transcriptional expression levels of EMT-related genes in HeLa cells treated with TGF-β1 for 24 h. Total RNA was extracted from TGF-β1-treated cells using TRIzol reagent and analyzed by RT-PCR for snail, slug, vimentin, and E-cadherin. GAPDH was used as a control. The histogram shows the results of ImageJ data analysis. Data are represented as the mean percentage of distance ± SD from at least three replicates, ** *p* < 0.01, * *p* < 0.05 for all experiments.

Previously we reported ROS-mediated EMT in TGF-β1-induced human cervical carcinoma (HeLa) cells. We also found that upon TGF-β1 treatment, among the NOX1–5 family, NOX2 and NOX4 were induced [28,29]. In this study, we hypothesized and confirmed that ROS might play a role in TGF-β1-induced EMT in HeLa cells through activation of the NOX pathway. TGF-β1 treatment for 24 h induced NOX2 and NOX4 expression at both the protein and mRNA level (Figure 1c,d). The induction of NOX2/4 led to the production of ROS, as detected by 2’,7’-dichlorofluorescein-diacetate (DCFDA) assays (Figure 1a). Moreover, we found that diphenyleneiodonium chloride (DPI) treatment could ameliorate ROS production, which confirmed that ROS were produced by NOX in our system (Figure 1b). Another important regulatory mechanism in the metastatic cascade involves the activation of cell migration. Scratch assays indicated that TGF-β1 treatment could accelerate cell motility; however, DPI inhibited the TGF-β1-mediated increase in cell motility indicating that this process is associated with ROS (Figure 1e).

We further examined whether ROS are also involved in EMT-related gene expression. As shown by Western blotting (Figure 1f) and RT-PCR (Figure 1g), the EMT-associated mesenchymal markers snail, slug, and vimentin were downregulated upon DPI treatment, whereas the adherens junction proteins E-cadherin and ZO-1 were significantly increased (Figure 1f,g). These results suggested that TGF-β1-mediated NOX2 and NOX4 activity is blocked by DPI, which effectively reverses TGF-β1-induced changes in EMT factors.

### 3.2. Distinct Pattern of NOX Isoforms’ Spatial-Temporal Expression in TGF-β1 Treated HeLa Cells

**Figure 2:**
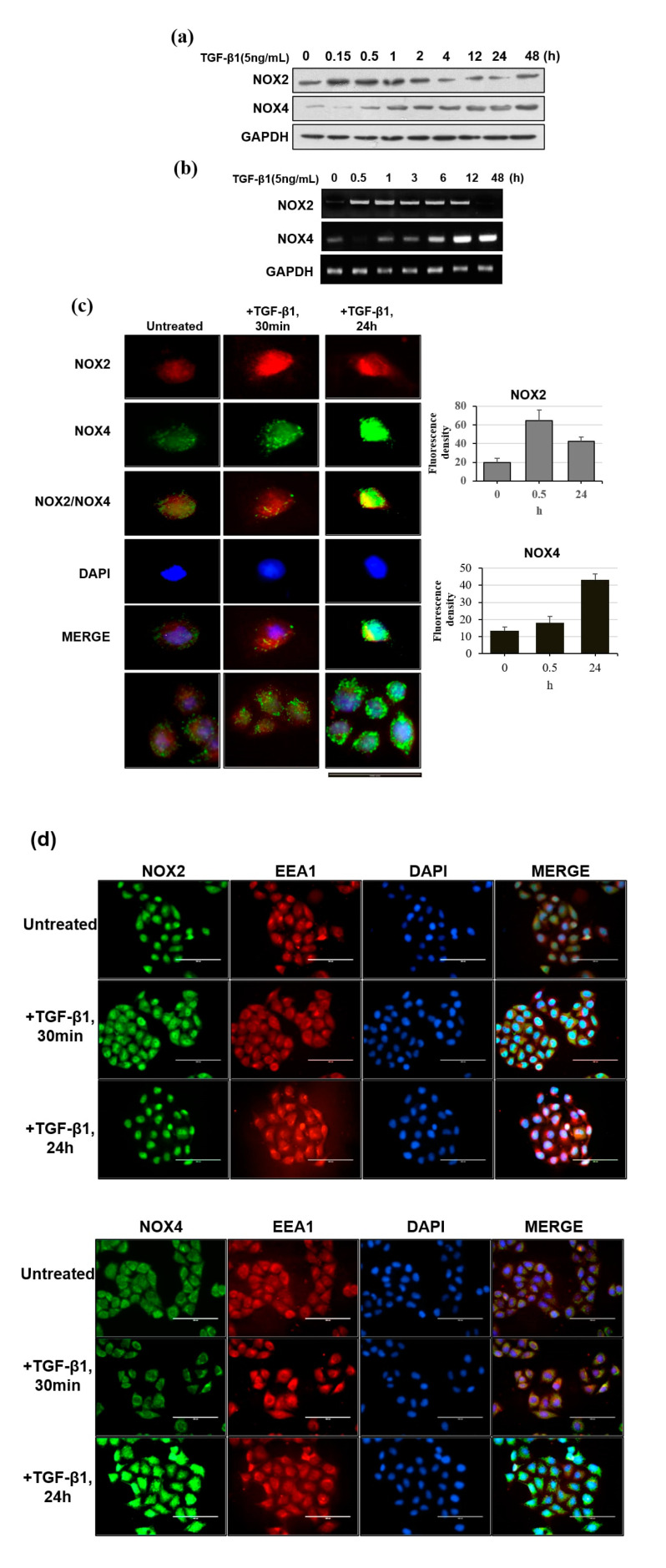

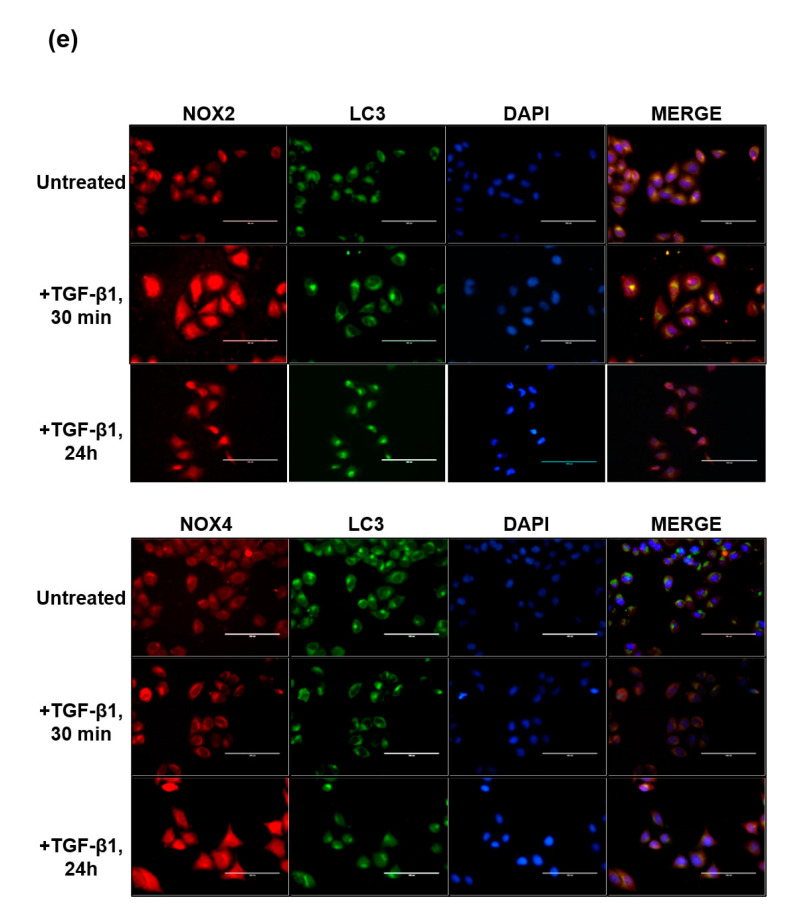
Changes in the expression of NOX2 and NOX4 in the presence of 5 ng/mL TGF-β1 for 48 h. (**a**) Representative Western blot depicting the time course (0.15, 0.5, 1, 2, 4, 12, 24, and 48 h) of NOX2 and NOX4 expression following TGF-β1 treatment. (**b**) HeLa cells were treated with TGF-β1 for 48 h. The expression of *NOX2* and *NOX4* was examined by RT-PCR. (**c**) TGF-β1-induced expression and determination of the intracellular co-localization of NOX2 and NOX4 in HeLa cells. NOX2 (red) and NOX4 (green) signals were merged with the cell nuclear dye DAPI (blue). NOX2 and NOX4 expression and intracellular location in untreated cells and cells treated with TGF-β1 for 30 min and 24 h cells are shown. The images were quantified using ImageJ. Generation of the graph and statistical analysis were carried out using Excel. ** *p* < 0.01, * *p* < 0.05. (**d**) Co-localization of NOX2 (green), NOX4 (green), and EEA1 (red) in cells treated with TGF-β1. Cells maintained in the presence or absence of TGF-β1 for 30 min or 24 h were visualized by immunocytochemistry. (**e**) Co-localization of NOX2 (red), NOX4 (red), and LC3 (green) in cells stimulated with TGF-β1 (0, 30 min, and 24 h). Nuclei were stained with DAPI. Scale bar in (c–e) represents 100 μm.

Based on the hypothesis that NOX2 and NOX4 have different roles, we examined their expression with TGF-β1 treatment over time. Weak basal expression of NOX2 and NOX4 proteins were observed in unstimulated cells, which progressively increased from 15 min to 48 h after the addition of TGF-β1. However, the expression of NOX2 and NOX4 exhibited different expression profiles over time upon TGF-β1 induction (Figure 2a,b). Specifically, NOX2 expression was initiated earlier and decreased as NOX4 expression increased in TGF-β1-stimulated HeLa cells.

We next determined whether differentially expressed NOX2 and NOX4 also exhibited distinct cellular localizations by performing co-localization immunohistochemistry using anti-NOX2 and anti-NOX4 antibodies. We found that the localization of NOX2 and NOX4 clearly differed between resting cells and TGF-β1-stimulated cells (Figure 2c). In support of our previous findings, the intensity of NOX2 was strong during the early period, whereas that of NOX4 was stronger at the late time point, as shown in Figure 2a,b.

Further, NOX2 exhibited primarily cytosolic distribution at the early time point whereas, NOX4 showed a novel punctuate pattern. Use of the early endosome marker EEA1 and autophagosome marker LC3 showed that NOX2 was not co-localized with either marker, but that NOX4 appeared to co-localize with LC3 (Figure 2d,e). These results indicated that upon TGF-β1 treatment, NOX2 and NOX4 were induced at different times and showed unique localization patterns.

### 3.3. NOX Regulated Redox Signaling Mechanisms

**Figure 3:**
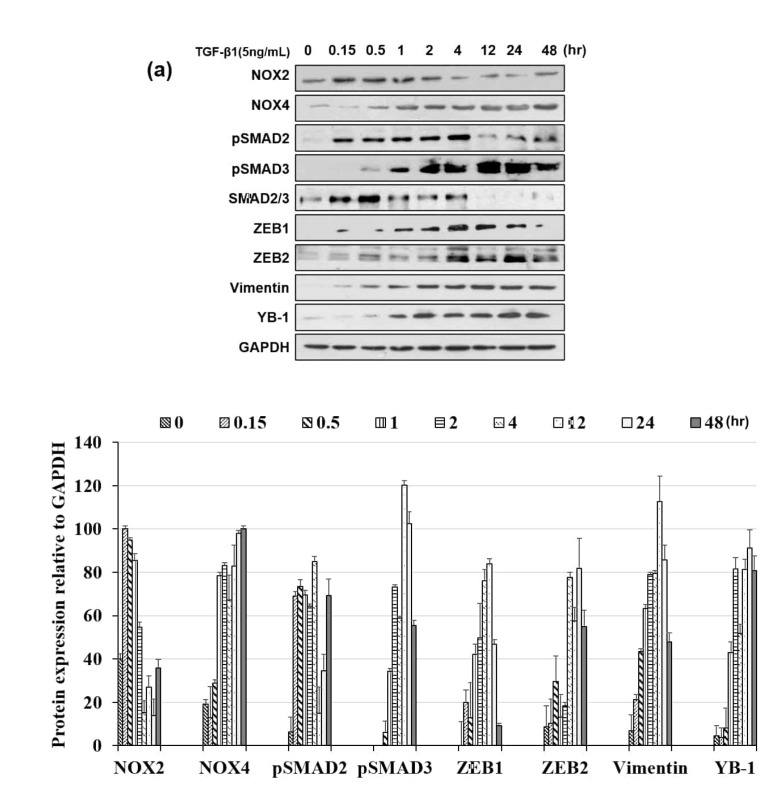

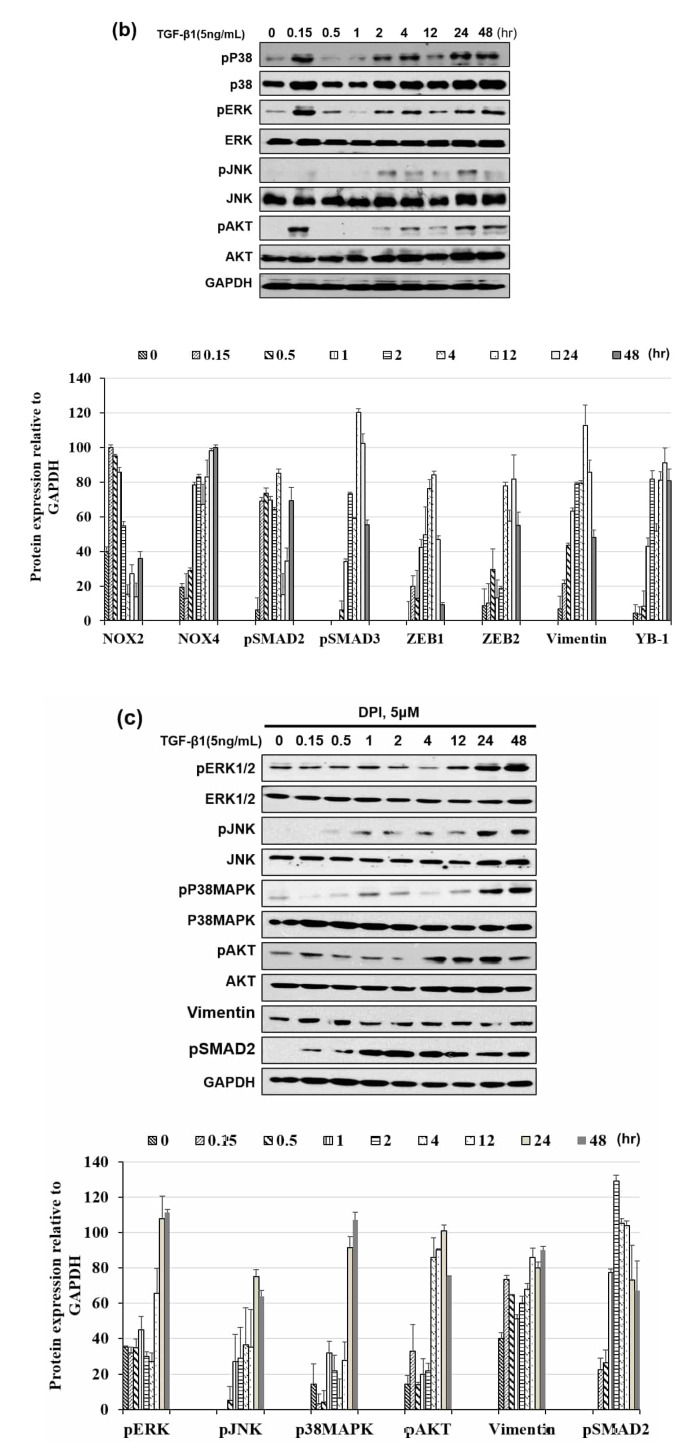

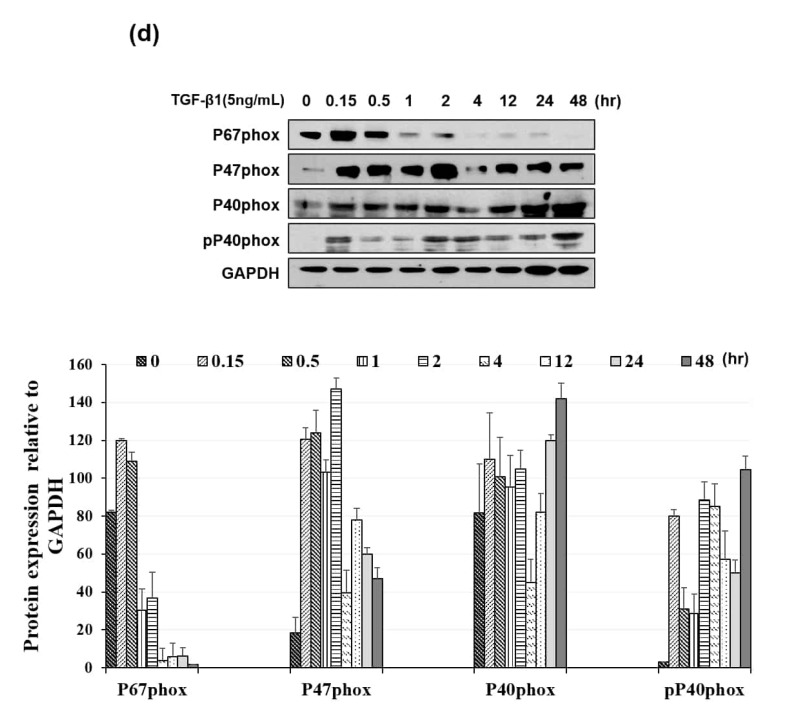
TGF-β1-induced activation of Smad-dependent and Smad-independent pathway proteins. (**a**) TGF-β1 (5 ng/mL) altered the expression of epithelial-to-mesenchymal transition (EMT) molecular markers. Cells were treated with TGF-β1 for 48 h. Total cell lysates were extracted for Western blot analysis of the expression of pSmad2/3, ZEB1/2, vimentin, and YB-1. GAPDH was used as a loading control. (**b**) Effects of TGF-β1 on phosphorylation of AKT, p38, and ERK1/2 MAPKs in a time-dependent manner. HeLa cells were treated with TGF-β1 for the indicated times. Cell lysates were analyzed by Western blotting with antibodies against phospho-Smad2/3, Smad2/3, phospho-Akt, Akt, phospho-Erk1/2, Erk1/2, phospho-p38, and p38MAPK. (**c**) Western blots showing the effects of DPI (5 μM) on TGF-β1-induced phosphorylation of AKT, ERK, and P38MAPK. Cells were treated with TGF-β1 for 48 h. (**d**) Expression of NADPH oxidase subunits including p22phox, p47phox, p67phox, and p40phox induced by TGF-β1. Assessment of NOX regulatory subunits p22phox, p40phox, p47phox, and p67phox by Western blot analysis. The GAPDH internal control protein was analyzed by Western blotting.

As changes in the redox state induced by TGF-β1 affect both canonical and non-canonical pathways, it is important to clarify the importance of downstream TGF-β1 effectors. Therefore, we tested the EMT transcription factors ZEB1, ZEB2, and vimentin, as well as Smad2/3. Notably, we found that Smad2 phosphorylation and the induction of ZEB1 and vimentin followed the NOX2 pattern over time, whereas Smad3 phosphorylation and ZEB2 induction followed that of NOX4 (Figure 3a). Moreover, another EMT inducer or regulator, YB-1, was induced according to the NOX4 activation pattern.

In testing non-canonical pathways, we found that the phosphorylation of p38MAPK, ERK, and AKT markedly increased within 10 min, decreased from 30 min to 1 h, and then were re-phosphorylated from 2 to 48 h. Conversely, no early surge in JNK phosphorylation was observed (Figure 3b). From Figure 3a,b, we could see that both canonical and non-canonical pathway signals were activated over NOX2 and NOX4 induction. We then treated cells with NOX inhibitor DPI and analyzed the responses. As shown in Figure 3c, canonical and non-canonical signals, except for Smad2, were ameliorated by DPI treatment, which suggests that apart from NOX2 and NOX4, involvement of other factors is involved in the activation of Smad to regulate the canonical and non-canonical pathways. Further, immediate activation of the non-canonical pathway upon TGF-β1 stimulation was very strong over NOX2 induction; therefore, we also examined other subunits of the NOX2 system. Specifically, P67phox and P47phox synthesis was induced and P40phox phosphorylation was detected within 10 min, which confirmed that early activation of the non-canonical pathway is mediated by NOX2 activation (Figure 3d). These results suggested that non-canonical signals (ERK, p38, and AKT) are activated by NOX2 at the early time point and later reactivated by NOX4.

### 3.4. Downregulating NOX2/4 Manifests Inhibition on Cell Motility and Proliferation

**Figure 4:**
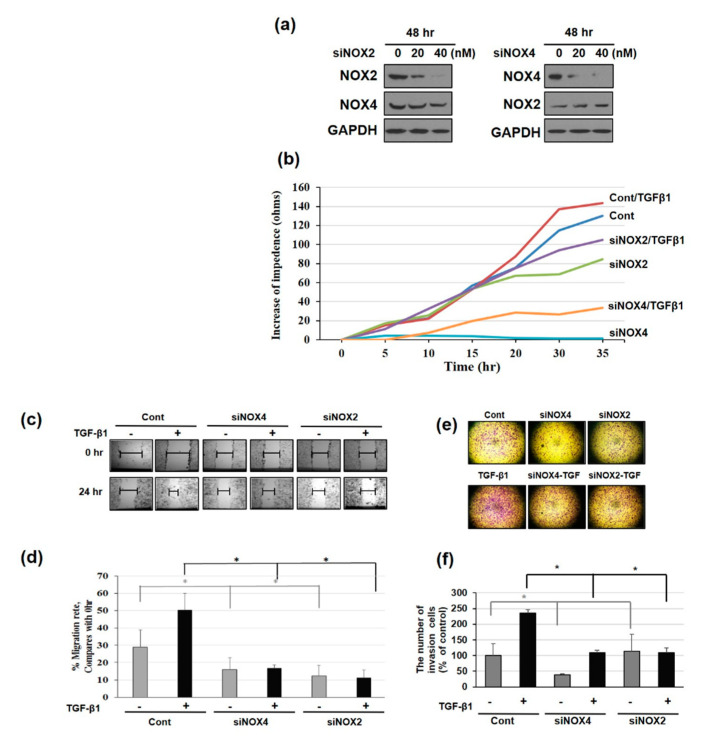
Effect of NOX2 or NOX4 knockdown on the proliferation, migration, and invasion of HeLa cells. (**a**) Representative Western blot of NOX2 and NOX4 protein expression in HeLa cells treated with siRNA-NOX2 or siRNA-NOX4 (at a final concentration of 20 or 40 nM) for 48 h. HeLa cells were transfected with NOX2 siRNA or NOX4 siRNA. After 24 h, cells were seeded over the electrode wells (**b**), six-well plate (**c**), and transwell plates (**d**). (**b**) HeLa cell growth curve in the presence of NOX2 or NOX4. NOX2 or NOX4 knockdown effect was assessed by measuring the change in HeLa cell growth responses using TGF-β1-treated cells by ECIS analysis over 35 h. (**c**,**d**) Inhibition of cell migration by NOX2 or NOX4 siRNA. (**c**) TGF-β1-induced cell migration was blocked by NOX2 or NOX4 siRNA. Cells were wounded by scratching the monolayer with a 200-μL sterile tip. The scratched monolayers were rinsed with PBS buffer and exposed to TGF-β1 for 24 h. (**d**) Quantitative analysis of the migration speed of cells. The distance traveled by the cell front was divided by the time-period to obtain the migration speed. After 24 h, the wound gaps were photographed using an inverted microscope and quantified by ImageJ software. Results shown are Control vs. NOX2-or NOX4-knockdown cells; * *p* < 0.0001, ** *p* < 0.00001, *n* = 10. Data are summarized as the means ± standard deviation of three separate experiments. *** *p* < 0.001 vs. control. (**e**) Invasive capacity of HeLa cells treated with NOX2 or NOX4 siRNA assessed using a Matrigel-coated transwell chamber. Transwell migration assay for negative control, TGF-β1-treated, and siRNA groups (magnification, ×400). The values represent the means ± SD; *n* = 3 based on microscopic fields. * *p* < 0.05.

Next, we downregulated NOX using siRNA treatment for 48 h and observed the effect on cell motility/proliferation. Inhibition of NOX expression was confirmed by Western blotting (Figure 4a). After 48 h of siRNA treatment followed by TGF-β1 addition, cell proliferation was observed for 35 h using an electric cell substrate impedance sensing (ECIS) system. Whereas NOX4 downregulation dramatically decreased control or TGF-β1-induced cell growth, NOX2 inhibition only slightly affected cell proliferation (Figure 4b). We next tested migration after NOX2 and NOX4 knockdown using scratch wound healing assays. NOX2 or NOX4 knockdown not only reduce cell migration but also attenuated TGF-β1-enhanced cell migration (Figure 4c,d), with relatively diminished migration in NOX2-knockdown cells. As cell migration is often affected by the rate of cell proliferation, two-chamber invasion assays were also performed (Figure 4e,f). Invasion was also decreased in both knockdown cell types, particularly TGF-β1 treatment could not augment invasion in NOX2-knockdown cells (Figure 4f). These results suggested that NOX4 knockdown affects cell proliferation to a greater degree, whereas NOX2 knockdown has a more marked effect on cell migration.

### 3.5. NOX4 Promotes Cell Proliferation Through Cyclin D and E Induction

**Figure 5:**
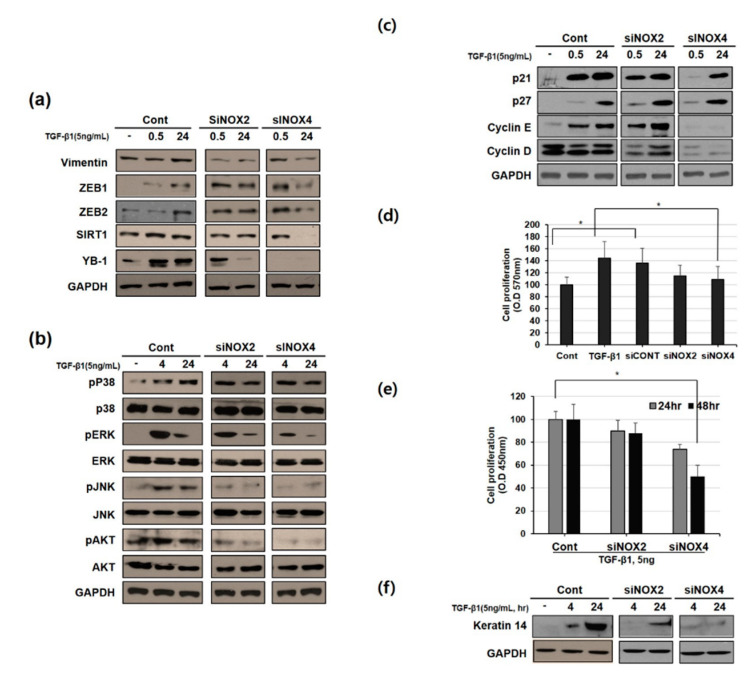
Inhibition of NOX2 or NOX4 suppresses TGF-β1-induced epithelial-to-mesenchymal transition (EMT). At 24 h post-transfection with NOX2 or NOX4 siRNA, the cells were treated with TGF-β1 (5 ng/mL) for an additional 24 h. (**a**) TGF-β1 altered the expression of EMT molecular markers. Western blots of mesenchymal markers including vimentin, ZEB1/2, Sirt1, and YB-1 in the indicated cells is shown in response to treatment with TGF-β1 for 0, 0.5, and 24 h. (**b**) Effects of TGF-β1 stimulation on HeLa cells. AKT, P38, ERK, and JNK were activated by TGF-β1 based on Western blot analysis of TGF-β1-stimulated HeLa cell protein extracts. (**c**) Western blot analysis of the indicated specific cyclins in NOX2- or NOX4-knockdown cells. (**d**,**e**) Effect of NOX2 or NOX4 knockdown by siRNA on cell proliferation. Cells were treated with TGF-β1 for 24 h and then cell viability was determined by performing MTT and BrdU assays. Values are expressed as the means ± standard deviation; *n* = 3, * *p* < 0.05, ** *p* < 0.01. (**f**) K14 levels in NOX2- or NOX4-knockdown and TGF-β1-treated cells, as analyzed by Western blotting. For (**a**–**e**), GAPDH was used as a loading control.

The time-dependent effect of NOX knockdown was further studied at early and late time points. Figure 5a shows that the expression of EMT markers such as vimentin, ZEB1/2, and YB-1, which followed the NOX4-induction time pattern (Figure 3a), was not affected by NOX2 knockdown but was rather inhibited at the later time point in NOX4-knockdown cells. The expression of sirtuin-1 deacetylase, which helps to modulate oxidative status in cells via FOXO3 deacetylation, and the induction of apoptosis were also decreased in NOX4-knockdown cells. Whereas total levels of proteins involved in non-canonical signaling cascades (AKT, JNK/P38, ERK) remained virtually constant in both control and NOX-knockdown cells, the activated forms (phosphorylated) of those signaling proteins were induced upon TGF-β1 treatment leading to EMT. However, NOX silencing (siNOX2 and siNOX4) reversed this effect by inhibiting the phosphorylation of ERK, JNK, and AKT, thereby blocking EMT (Figure 5b). Knockdown of NOX2 did not influence the cell cycle inhibitors p21 and p27 or cell cycle progression-related proteins cyclin E and D (Figure 5c). However, NOX4 knockdown inhibited the expression of cyclin E and D. This motivated us to analyze cell proliferation after NOX silencing by performing MTT and BrdU assays. MTT assays showed that NOX2 or NOX4 downregulation significantly inhibited cell growth at 24 and 48 h in TGF-β1-treated HeLa cells. Further studies based on BrdU assays revealed that NOX4 knockdown significantly inhibited BrdU incorporation (Figure 5d,e). Moreover, NOX2 or NOX4 siRNA transfection significantly decreased the expression of keratin 14 (K14) compared to that in TGF-β1 treated cells (Figure 5f).

### 3.6. Focal Adhesion Kinase and SRC Phosphorylation are Involved in TGF-β1-Mediated Cell Migration Via NOX2 Activation

**Figure 6:**
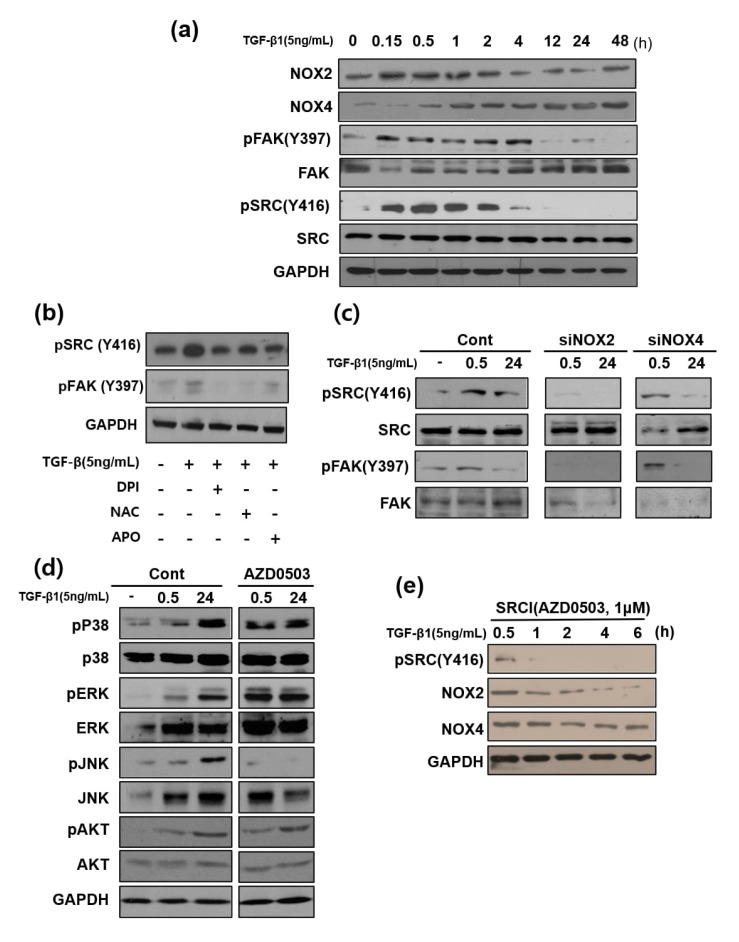

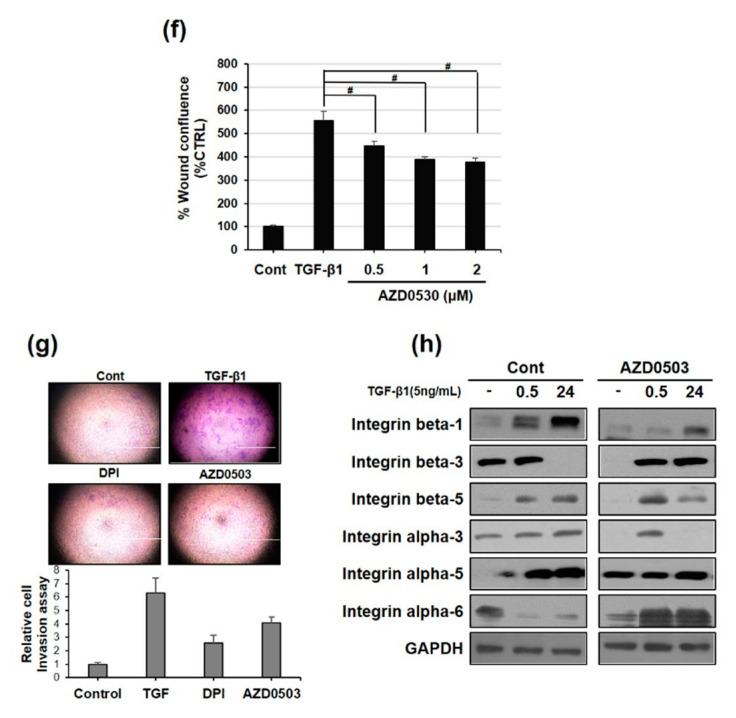
TGF-β1 induces Src activation, and AZD0530 inhibits TGF-β1-induced Src activation in a time-dependent manner in HeLa cells. (**a**) Expression of focal adhesion kinase (FAK), Src, and their phosphorylation after TGF-β1 (5 ng/mL) treatment for different times in HeLa cells, as measured by Western blot analysis. (**b**) Effect of NOX and a reactive oxygen species (ROS) inhibitor on TGF-β1-stimulated phosphorylation of SRC and FAK. After pre-incubation for 1 h with inhibitors, cells were grown in the presence of TGF-β1 for 6 h and then analyzed. (**c**) Cells were transfected with NOX2 or NOX4 siRNA for 0, 30 min, and 24 h. After 24 h, total extracts were obtained. The content of SRC (Y416) and FAK (Y379) was assessed by Western blotting. (**d**) Activation of MAPK and Akt in response to TGF-β1 signaling. HeLa cells were exposed to TGF-β1. After 0, 0.5, and 24 h, HeLa cells were pre-incubated with AZD0530 (a SRC inhibitor) for 1 h. After TGF-β1 was added for 0, 0.5, and 24 h, phospho-ERK/ERK, phospho-p38MAPK/p38MAPK, phospho-JNK/JNK, the total form of each MAPK, and Src levels were analyzed by Western blotting. (**e**) The Src inhibitor AZD0530 (50 nM) was added 1 h before stimulation with TGF-β1. NOX2 and NOX4 protein expression in TGF-β1-treated cells at 0.5, 1, 2, 4, and 6 h is shown. (**f**,**g**) Migratory capacity of cells before treatment with AZD0530 for 24 h was analyzed by migration (f) and transwell assays (g). The invasive capacity of the cells was analyzed using Transwell filter chambers coated with Matrigel. (**h**) AZD0530 inhibits TGF-β1-induced integrin expression. Results show that TGF-β1 regulates integrin protein expression and indicate the inhibition of integrin by a SRC inhibitor. GAPDH expression was included as a loading control. # represents that the experimental outcome is statistically significant.

We next investigated the cell migration-related proteins Src and FAK. The TGF-β1-induced time-dependent peak in Src and FAK (Y397) phosphorylation continued for up to 4 h. Time course observation of Src and FAK activation revealed that their phosphorylation followed the NOX2-activation time pattern (Figure 6a). To confirm that phosphorylation was triggered by ROS generated by NOX2 and NOX4, the general ROS scavenger N-acetyl-L-cysteine (NAC) and several NOX inhibitors (DPI and apocynin) were applied along with TGF-β1, which resulted in inhibited FAK and Src phosphorylation (Figure 6b). Notably, FAK and Src phosphorylation were inhibited in the presence of TGF-β1 only in NOX2-knockdown cells (Figure 6c). Src inhibitor treatment did not affect non-canonical signaling, except JNK phosphorylation, at the later time point (Figure 6d). In addition, among non-canonical signals examined (Figure 3b), only JNK did not show an early activation pattern. This result suggests that the activation of JNK at the later time point does not occur via the same mechanism. Notably, treatment with the Src inhibitor AZD0503 along with TGF-β1 abolished NOX2 induction after 1 h but did not affect NOX4 induction. In addition, early Src phosphorylation was detected at 30 min (Figure 6e). We consider that TGF-β1 activated NOX2, with Src activation subsequently activating NOX2 induction in a feed-forward manner. Src inhibitor treatment also ameliorated TGF-β1-induced cell migration in a dose-dependent manner based on scratch wound assays (Figure 6f). Similarly, based on cell invasion assays, Src inhibition also reduced TGF-β1-induced cell invasion (Figure 6g).

Additionally, TGF-β1 activated the expression of integrin alpha-5, beta-1, and beta-5 and repressed integrin alpha-6 and beta-3. In comparison, upon treatment with AZD0530, an inhibitor of Src activation, levels of integrin beta 1 and alpha 3 were significantly decreased and those of integrin beta 3 and alpha 6 were increased (Figure 6h), which indicated that Src activation mediated by TGF-β1 was likely involved in these changes.

## 4. Discussion

Redox biology involving NOX has prominent role in diverse cellular events associated with epithelial malignancy such as EMT, angiogenesis, apoptosis evasion, and enhancement of metastatic potential [30]. Despite similarities in core structures, NOX homologues have different mechanisms of activation [7]. NOX2 depends on p22phox, the activation of which requires assembly with the cytosolic regulatory subunits p47phox, p67phox, and p40phox; in contrast, NOX4 does not require cytosolic activator subunits. The induction of NOX4 gene expression by TGF- β1 is Smad3-dependent, whereas this effect is strongly counteracted by wild-type p53 in breast cancer cells [31]. Recent studies have also demonstrated that NOX4 plays an important role in melanoma and urothelial carcinoma cell proliferation by regulating cell cycle progression [32,33]. Prior assessment of NOX complex localization indicated that both NOX2 and NOX4 are present in intracellular compartments or plasma membranes [34]. NOX2 is localized to the plasma membrane, phagosomes, endosomes, and the leading edge of lamellipodia, whereas NOX4 is localized to the plasma membrane, focal adhesions, nucleus, mitochondria, and endoplasmic reticulum in primary human endothelial cells [35]. In this present study, we showed that induction of NOX isoforms is time- and cellular localization-specific with TGF-β1 driven EMT related pathological cytoskeletal remodeling towards cervical cancer progression and involves tyrosine phosphorylation of FAK/SRC kinase as its downstream signaling cascade thereby aiding NOX related EMT events. TGF-β1 treatment resulted in upregulation of NOX2/4 and rapid intracellular production of ROS, which coincides with procurance of cancer associated cellular characteristics such as enhanced cell migration, proliferation, invasion, etc. Downregulating NOX through siRNA treatment demonstrated that early induced NOX2 promoted cell motility, while the delayed NOX4 induction drives cell proliferation upon cytokine induction.

Low doses of hydrogen peroxide and superoxide stimulate cell proliferation in a wide variety of cancer cell types [2,3,4,5]. In addition, ROS can upregulate the mRNA levels of cyclins, which participate in the cell cycle to expedite G1 to S phase transition, including cyclin B2/D3/E1/E2 [36]. The treatment of MCF-10A cells with the antioxidant NAC was found to induce delays in G1 to S progression, accompanied by a decrease in cyclin D1 levels [37]. K14-knockdown cells also exhibit reduced cell proliferation and delayed cell cycle progression, concomitant with Akt phosphorylation levels [38]. In the present study, NOX4-mediated signaling was found to be an important regulator of cell cycle-controlling events. Silencing NOX4 downregulated the expression of Cyclin D and Cyclin E, both of which has essential role in regulating cell cycle transition from G1/S phase. Further, cytokine induced K14 expression was found to be reduced with the silencing of NOX isoforms.

Src (a non-receptor tyrosine kinase) and FAK (a cytoplasmic tyrosine kinase) plays vital role in cell spreading, proliferation, differentiation, metastasis, cell cycle regulation, etc., without which cells find it difficult to align cytoskeletal polarity and organization [23]. While the role of Src kinase in cancer advancement is an extensively known observation, the involvement of redox signaling to activate Src in these pathological conditions is less explored. Herein, we observed that NOX2-induced ROS play an important role in cell migration during TGF-β1-induced EMT by regulating SRC and FAK phosphorylation. Our data regarding NOX2 and Src also suggest that Src has important roles upstream and downstream of ROS. As Src-family kinases are said to be involved in integrin-mediated cell adhesion, we observed that inhibiting Src phosphorylation affected the expression of several integrin molecules required for EMT progression. The activity of β1 and β5-integrins and integrin-linked kinase are required for TGF-β1-induced EMT in mammary epithelial cells. In addition, β5-integrins is said to contribute to cell invasion in breast carcinoma cells [39,40]. TGF-β1 is said to control the expression of av, β3, and β1 integrin subunits through both canonical and non-canonical pathways [41,42]. A recent study demonstrated that β1 and β3 integrin mediate TGF-β-induced EMT via Src signaling [24,43]. Further, the mesenchymal marker ZEB2 has been shown to activate integrin α5 and vimentin leading to EMT [44,45]. Taken together with our results, an interaction between integrin-mediated downstream signaling and Src in the TGF-β1 induced EMT signaling pathway is evident and sequential blockade of Src is found to revert EMT associated cell migration, invasion, and extra-cellular matrix deposition.

## 5. Conclusions

In this study, we provide evidence that TGF-β1-induced HeLa cell growth and migration are differentially and selectively controlled by NOX2 and NOX4. While silencing NOX4 diminishes TGF-β1-induced cell proliferation, NOX2 silencing reverts TGF-β1-induced cell motility and migration. Further, NOX drives Src/FAK kinase phosphorylation and intervention using Src inhibitor reverts back the NOX induced EMT cellular events. The question therefore arises how NOX isotypes differentially influence the fate of HeLa cells upon TGF-β1-induced EMT. One possible mechanism is related to the nature of ROS being generated by the two isoforms. Notably, NOX2 is known to generate superoxide anion radicals that mediate single electron signaling, whereas NOX4 is known to generate hydrogen peroxide, which mediates dual electron signaling [46]. Another potential mechanism is the different localization patterns of these ROS-generating enzymes. Thus, identifying the type, relative concentration, and tissue- and cell-specific locations of NOX homologs could provide more detailed insight into differences in cell proliferation and migration patterns observed during EMT. Together, our findings suggest that NOX2 and NOX4 play different roles in TGF-β1-driven HeLa cell proliferation and migration.

## Figures and Tables

**Table 1:** Specific primers.

Primer	Forward (5′→3′)	Reverse (5′→3′)
NOX2	CAACCTGGAAGGCTACCACT	CCTCATTCACAGCGCAGT
NOX4	CCAAGCAGGAGAACCAGGAG	GCAACGTCAGCAGCATGTAG
Snail	GAGGACAGTGGGAAAGGCTC	TGGCTTCGCATGTGCATCTT
Slug	GAACTCACACGGGGGAGAAG	ACACAGCAGCCAGATTCCTC
Vimentin	AATGGCTCGTCACCTTCGTGAAT	CAGATTAGTTTTCCCTCAGGTTCAG
E-cadherin	CCTGGGACTCCACCTACAGA	GGATGACACAGCGTGAGAGA
GAPDH	GAGAAGGCTGGGGCTCATTT	ACTGATGGCATGGACTGTGG

## Abbreviations

BrdU	5-bromodeoxyuridine
DCFDA	2′, 7′-dichlorfluorescein-diacetate
DMEM	Dulbecco’s modified Eagle medium
DPI	diphenyleneiodonium chloride
ECIS	electric cell substrate impedance sensing
ECM	extracellular matrix
EMT	epithelial–mesenchymal transition
FAK	focal adhesion kinase
FBS	fetal bovine serum
K14	keratin 14
MTT	3-(4,5-dimethyl-2-thiazolyl)-2,5-diphenyl-2H-tetrazolium bromide
NOX	NADPH oxidase
PBS	phosphate-buffered saline
ROS	reactive oxygen species
RT-PCR	reverse transcription-polymerase chain reaction
NAC	N-acetyl-l-cysteine
